# Department of Therapeutics

**Published:** 1894-05

**Authors:** 

**Affiliations:** Demonstrator of Physiology and Lecturer on the History of Medicine in the Medical Department of the University of Texas, etc.; Galveston


### Current Medical Literature.

### DEPARTMENT OF THERAPEUTICS.

EDITED BY DAVID CERNA, M. D., PH. D.,
Demonstrator of Physiology and Lecturer on the History of Medicine in the Medical Department of the University of Texas, etc.

### THE EXTERNAL USES OF GUAIACOL.

In the *Province Mêdicale* for February 3rd there is an article on this subject of which the following is the substance: This procedure, which was used by Sciolla and Bard, has for some time been employed as an antithermic. It consists in painting the greater part of the exterior wall of the thorax, and sometimes the forearms, with pure guaiacol. According to different authors the doses are variable, ranging from one to two cubic centimeters up to seven or eight. There is a difference of opinion as to the employment of this liquid. Sociolla, Bard, and other physicians, use pure guaiacol, while others, like Desplats, mix it with glycerine or alcohol. In several cases of advanced phthisis, a marked reduction of 2° has been obtained by painting the entire surface of the front of the thorax with pure guaiacol. Unfortunately the effects of this treatment are only temporary, not lasting more than three or four days. Sometimes, also, applications of this kind produce a marked rise in temperature—in one case of 2°. It is necessary, then, in making use of this procedure to ascertain the susceptibility of the patient, and to use at first small and then progressively large doses. There have been of late years interesting attempts made in the employment of this procedure. Casasovici and Miron Sigalea have used guaiacol mixed with tincture of iodine, in the treatment of pleurisy, in the following proportion: Tincture of iodine, three hundred and eighty-five grains; guaiacol, seventy-five grains. This quantity is used in a single application, and the diseased parts are thoroughly and extensively painted with it every night. These applications cause a considerable reduction of temperature, profuse perspiration, and increase the flow of urine, followed soon after by complete resorption. These results seem to have been obtained, particularly in one case, where there was abundant pleuritic effusion on the left side, in which tapping had not been followed by any relief, but had caused considerable rise of temperature, by the application of iodized guaiacol, the fever disappearing in a few days, and the effusion becoming resorbed. M. Desplats has recently conceived the idea of applying guaiacol in the treatment of painful rheumatic inflammation of the joints, after having observed a case in which applications of guaiacol had been used with excellent results. He has used a mixture of equal parts of guaiacol and pure glycerine. The joints were thoroughly painted with this mixture and afterwards covered with a dry dressing. In one case of acute rheumatism, and in three others of arthritis deformans, with sharp pains, the results were excellent. The pain was completely subdued, and in the first case the patient recovered rapidly. This procedure has recently been employed in applying guaiacol for articular neuralgia of the shoulder, which was very painful, in a tuberculous patient, who experienced marked relief. It is easily employed and not dangerous, if the indications mentioned are conformed to.—*N. Y. Medical Journal, March 3rd, 1894.*

